# Immunogenicity of a DNA vaccine candidate for COVID-19

**DOI:** 10.1038/s41467-020-16505-0

**Published:** 2020-05-20

**Authors:** Trevor R. F. Smith, Ami Patel, Stephanie Ramos, Dustin Elwood, Xizhou Zhu, Jian Yan, Ebony N. Gary, Susanne N. Walker, Katherine Schultheis, Mansi Purwar, Ziyang Xu, Jewell Walters, Pratik Bhojnagarwala, Maria Yang, Neethu Chokkalingam, Patrick Pezzoli, Elizabeth Parzych, Emma L. Reuschel, Arthur Doan, Nicholas Tursi, Miguel Vasquez, Jihae Choi, Edgar Tello-Ruiz, Igor Maricic, Mamadou A. Bah, Yuanhan Wu, Dinah Amante, Daniel H. Park, Yaya Dia, Ali Raza Ali, Faraz I. Zaidi, Alison Generotti, Kevin Y. Kim, Timothy A. Herring, Sophia Reeder, Viviane M. Andrade, Karen Buttigieg, Gan Zhao, Jiun-Ming Wu, Dan Li, Linlin Bao, Jiangning Liu, Wei Deng, Chuan Qin, Ami Shah Brown, Makan Khoshnejad, Nianshuang Wang, Jacqueline Chu, Daniel Wrapp, Jason S. McLellan, Kar Muthumani, Bin Wang, Miles W. Carroll, J. Joseph Kim, Jean Boyer, Daniel W. Kulp, Laurent M. P. F. Humeau, David B. Weiner, Kate E. Broderick

**Affiliations:** 1 Inovio Pharmaceuticals, Plymouth Meeting, Philadelphia, PA 19462 USA; 2 Vaccine and Immunotherapy Center, Wistar Institute, Philadelphia, PA 19104 USA; 3 National Infection Service, Public Health England, Porton Down, Wiltshire, UK; 4 Advaccine (Suzhou) Biopharmaceuticals Co., Ltd, Suzhou, China; 5 Key Laboratory of Medical Molecular Virology of MOH and MOE and Department of Medical Microbiology and Parasitology, School of Basic Medical Sciences, Fudan University, Shanghai, China; 6 Department of Molecular Biosciences, The University of Texas at Austin, Austin, TX 78712 USA

**Keywords:** Cellular immunity, Antibodies, DNA vaccines, SARS-CoV-2

## Abstract

The coronavirus family member, SARS-CoV-2 has been identified as the causal agent for the pandemic viral pneumonia disease, COVID-19. At this time, no vaccine is available to control further dissemination of the disease. We have previously engineered a synthetic DNA vaccine targeting the MERS coronavirus Spike (S) protein, the major surface antigen of coronaviruses, which is currently in clinical study. Here we build on this prior experience to generate a synthetic DNA-based vaccine candidate targeting SARS-CoV-2 S protein. The engineered construct, INO-4800, results in robust expression of the S protein in vitro. Following immunization of mice and guinea pigs with INO-4800 we measure antigen-specific T cell responses, functional antibodies which neutralize the SARS-CoV-2 infection and block Spike protein binding to the ACE2 receptor, and biodistribution of SARS-CoV-2 targeting antibodies to the lungs. This preliminary dataset identifies INO-4800 as a potential COVID-19 vaccine candidate, supporting further translational study.

## Abstract (web summary)

There is currently no licensed SARS-CoV-2 vaccine. Here, the authors generate an optimized DNA vaccine candidate encoding the SARS-CoV-2 spike antigen, demonstrating induction of specific T cells and neutralizing antibody responses in mice and guinea pigs. These initial results support further development of this vaccine candidate.

## Introduction

COVID-19, known previously as 2019-nCoV pneumonia or disease, has emerged as a global public health crisis, joining severe acute respiratory syndrome (SARS) and Middle East respiratory syndrome (MERS) in a growing number of coronavirus-associated illnesses which have jumped from animals to people. There are at least seven identified coronaviruses that infect humans. In December 2019 the city of Wuhan in China became the epicenter for an outbreak of the novel coronavirus, SARS-CoV-2. SARS-CoV-2 was isolated and sequenced from human airway epithelial cells from infected patients^1,2^. Disease symptoms range from mild flu-like to severe cases with life-threatening pneumonia^3^. The global situation is dynamically evolving, and on 30 January 2020 the World Health Organization declared COVID-19 as a public health emergency of international concern (PHEIC), and on 11 March 2020 it was declared a global pandemic. As of 1 May 2020 there are 3,321,402 people confirmed infected and 237,180 deaths^4^. Infections have spread to multiple continents. Human-to-human transmission has been observed in multiple countries, and a shortage of disposable personal protective equipment^5^, and prolonged survival times of coronaviruses on inanimate surfaces^6^, have compounded this already delicate situation and heightened the risk of nosocomial infections. Advanced research activities must be pursued in parallel to push forward protective modalities in an effort to protect billions of vulnerable individuals worldwide. Currently, no licensed preventative vaccine is available for COVID-19.

To address the urgent need for a medical countermeasure to prevent the further dissemination of SARS-CoV-2 we have employed a synthetic DNA-based vaccine approach. Synthetic DNA vaccines are amenable to accelerated developmental timelines due to the ability to quickly design multiple candidates for preclinical testing, scalable manufacturing of large quantities of the drug product, and the possibility to leverage established regulatory pathways to the clinic. Synthetic DNA is temperature-stable and cold-chain free, important features for delivery to resource-limited settings^7^. Specifically for the development of a COVID-19 vaccine candidate, we leveraged prior experiences in developing vaccine approaches to SARS-CoV^8^, and our own experience in developing a MERS-CoV vaccine (INO-4700)^9,10^, as well as taking advantage of our vaccine design and manufacturing pathway previously utilized for the Zika vaccine candidate, GLS-5700^11^, which was advanced to the clinic in under 7 months. INO-4700 and GLS-5700 vaccines are currently in clinical testing.

Prior work has demonstrated that a DNA approach for SARS and MERS can drive neutralizing antibody (nAb) responses and provide protection in challenge models^8,10^. Our previous studies indicated immunization of small and large animal models with DNA vaccines encoding MERS-CoV spike (S) protein provided protection against disease challenge with the matched virus. In subjects immunized with INO-4700 (MERS-CoV S protein DNA vaccine) durable neutralizing antibodies (nAbs) and T cell immune responses were measured, and a seroconversion rate of 96% was observed and immunity was followed for 60 weeks in most study volunteers^9^. INO-4700 Phase 1/2a testing is continuing in South Korea, and a larger Phase 2 study is being planned to begin in the Middle East, both areas which have been most affected by MERS infections.

**Fig. 1:**
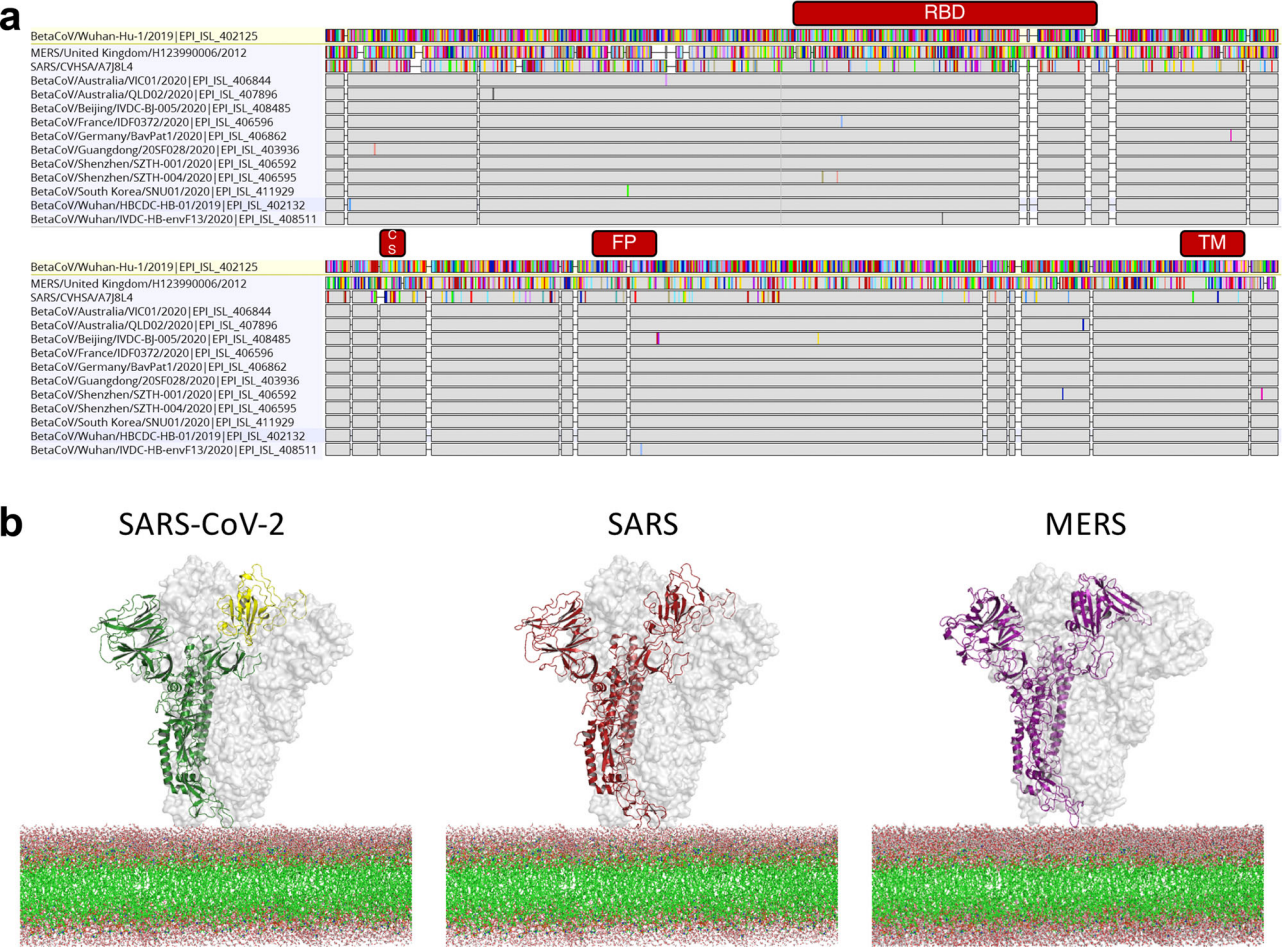
Comparison of SARS-CoV-2, SARS-CoV and MERS-CoV spike glycoproteins. **a** Amino acid alignment of coronavirus spike proteins including 11 SARS-CoV-2 sequences with mutations (GISAID). Gray bars indicates identical amino acids and colored bars represent mutations relative to Wuhan-Hu-1. RBD, Cleavage Site, Fusion Peptide and Transmembrane domains are indicated in red. **b** Structural models for SARS-CoV-2, SARS and MERS spike glycoproteins with one chain represented as cartoon and two chains represented as surface. RBD of SARS-CoV-2 is colored yellow.

The SARS-CoV-2 spike is most similar in sequence and structure to SARS-CoV spike protein^12^, and shares a global protein fold architecture with the MERS-CoV spike protein (Fig. 1) allowing us to build on our prior vaccine construct design^10^. Unlike glycoproteins of HIV and influenza, the prefusion form of the coronavirus trimeric spike is conformationally dynamic, fully exposing the receptor-binding site infrequently^13^. The receptor-binding site is a vulnerable target for nAbs. In fact, MERS nAbs targeted at the receptor-binding domain (RBD) tend to have greater neutralizing potency than other epitopes^14^. A recent report demonstrated that an anti-SARS antibody could cross-react to the RBD of SARS-CoV-2^15^. These data suggest that the SARS-CoV-2 RBD is an important target for vaccine development. Recent data has revealed SARS-CoV-2 S protein binds the same host receptor, angiotensin-converting enzyme 2 (ACE2), as SARS-CoV S protein^12^.

Here, we describe the design and initial preclinical testing of COVID-19 synthetic DNA vaccine candidates. We show the expression of the SARS-CoV-2 S antigen RNA and protein after in vitro transfection of COS-7 and 293T cells, respectively, with the vaccine candidates. We followed the induction of immunity by the selected immunogen in mice and guinea pigs, measuring SARS-CoV-2 S protein-specific antibody levels in serum and in the lung fluid, and antibody functionality through competitive inhibition of ACE2 binding, pseudovirus and live virus neutralization. The INO-4800 vaccine induces cellular and humoral host immune responses that can be observed within days following a single immunization, including cross-reactive responses against SARS-CoV. The data demonstrate the immunogenicity of this COVID-19 synthetic DNA vaccine candidate targeting the SARS-CoV-2 S protein, supporting further translational studies to advance the development of this candidate in response to the current global health crisis.

## Results

### Design and synthesis COVID-19 DNA vaccine constructs

**Fig. 2:**
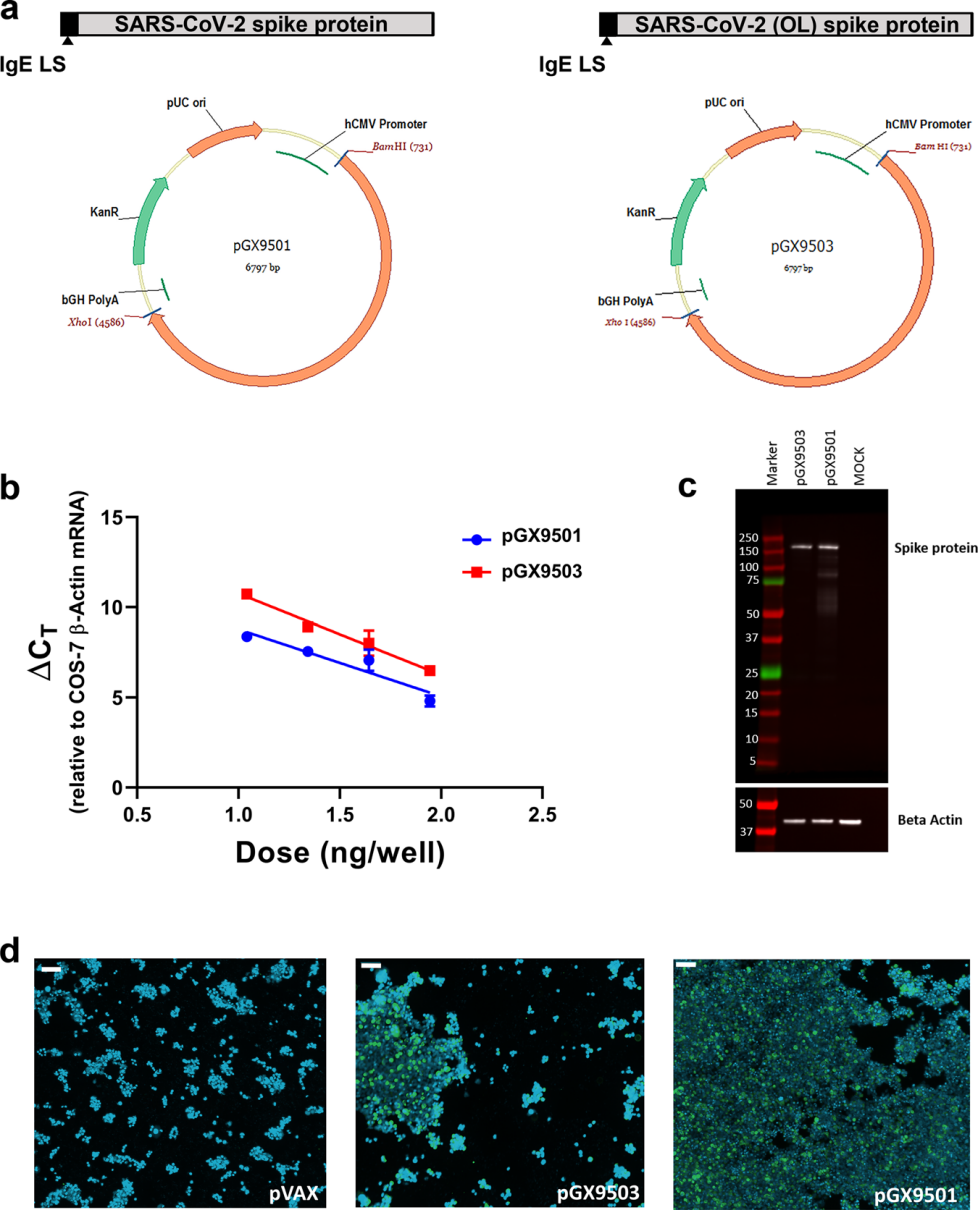
Design and expression of COVID-19 synthetic DNA vaccine constructs. **a** Schematic diagram of COVID-19 synthetic DNA vaccine constructs, pGX9501 (matched) and pGX9503 (outlier (OL)) containing the IgE leader sequence and SARS-CoV-2 spike protein insert. **b** RT-PCR assay of RNA extracts from COS-7 cells transfected in duplicate with pGX9501 and pGX9503. Extracted RNA was analyzed by RT-PCR using PCR assays designed for each target and for COS-7 β-Actin mRNA, used as an internal expression normalization gene. Delta C_T_ (∆ C_T_) was calculated as the C_T_ of the target minus the C_T_ of β-Actin for each transfection concentration and is plotted against the log of the mass of pDNA transfected (Plotted as mean ± SD). **c** Analysis of in vitro expression of Spike protein after transfection of 293T cells with pGX9501, pGX9503 or MOCK plasmid by Western blot. 293T cell lysates were resolved on a gel and probed with a polyclonal anti-SARS Spike Protein. Blots were stripped then probed with an anti-β-actin loading control. **d** In vitro immunofluorescent staining of 293T cells transfected with 3 µg/well of pGX9501, pGX9503 or pVax (empty control vector). Expression of Spike protein was measured with polyclonal anti-SARS Spike Protein IgG and anti-IgG secondary (green). Cell nuclei were counterstained with DAPI (blue). Images were captured using ImageXpress Pico automated cell imaging system. Scale bars are 80.15 µm (left), 66.8 µm (middle) and 77.31 µm (right).

Four spike protein sequences were retrieved from the first four available SARS-CoV-2 full genome sequences published on GISAID (Global Initiative on Sharing All Influenza Data). Three Spike sequences were 100% matched and one was considered an outlier (98.6% sequence identity with the other sequences). After performing a sequence alignment, the SARS-CoV-2 spike glycoprotein sequence was generated and an N-terminal IgE leader sequence was added. The highly optimized DNA sequence encoding SARS-CoV-2 IgE-spike was created using Inovio’s proprietary in silico Gene Optimization Algorithm to enhance expression and immunogenicity. The optimized DNA sequence was synthesized, digested with *BamHI* and *XhoI*, and cloned into the expression vector pGX0001 under the control of the human cytomegalovirus immediate-early promoter and a bovine growth hormone polyadenylation signal. The resulting plasmids were designated as pGX9501 and pGX9503, designed to encode the SARS-CoV-2 S protein from the three-matched sequences and the outlier sequence, respectively (Fig. 2a).

### In vitro characterization of COVID-19 DNA vaccine constructs

We measured the expression of the encoded SARS-CoV-2 spike transgene at the RNA level in COS-7 cells transfected with pGX9501 and pGX9503. Using the total RNA extracted from the transfected COS-7 cells we confirmed expression of the spike transgene by RT-PCR (Fig. 2b). In vitro spike protein expression in HEK-293T cells was measured by Western blot analysis using a cross-reactive antibody against SARS-CoV S protein on cell lysates. Western blots of the lysates of HEK-293T cells transfected with pGX9501 or pGX9503 constructs revealed bands approximate to the predicted S protein molecular weight, 140–142 kDa, with slight shifts likely due to the 22 potential N-linked glycans in the S protein (Fig. 2c). In immunofluorescent studies the S protein was detected in 293T cells transfected with pGX9501 or pGX9503 (Fig. 2d). In summary, in vitro studies revealed the expression of the Spike protein at both the RNA and protein level after transfection of cell lines with the candidate vaccine constructs.

### Humoral immune responses in mice

**Fig. 3:**
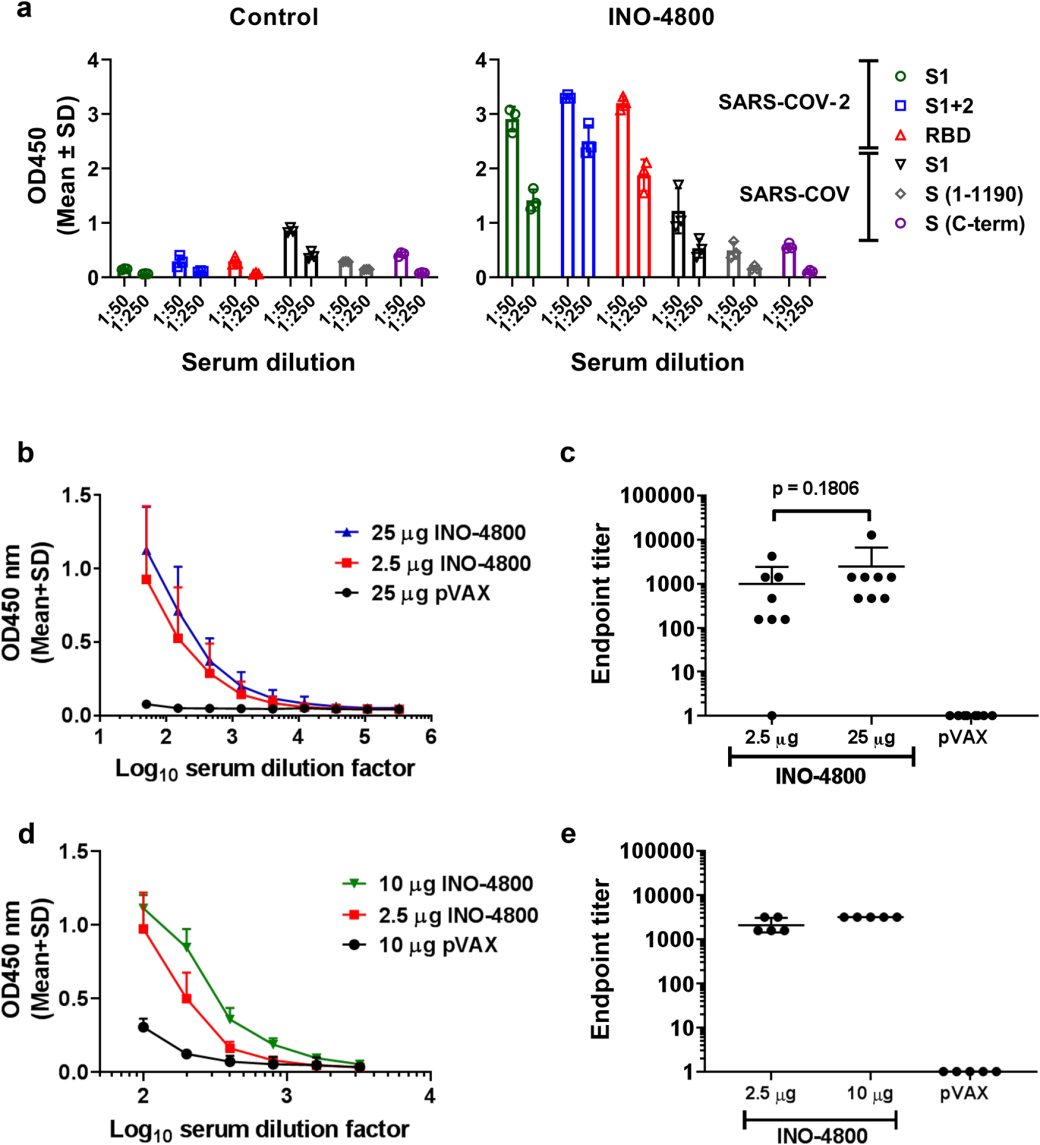
Humoral responses to SARS-CoV-2 and SARS-CoV antigens in BALB/c mice after a single dose of INO-4800. BALB/c mice were immunized on day 0 with indicated doses of INO-4800 or pVAX-empty vector as described in the methods. **a** Protein antigen binding of IgG at 1:50 and 1:250 serum dilutions from mice at day 14 immunized with 25 µg of INO-4800 or pVAX. Data shown represent mean OD450 nm values (mean + SD) for each group of 3 mice. **b** SARS-CoV-2 S1 + 2 or **c** SARS-CoV-2 RBD protein antigen binding of IgG in serial serum dilutions from mice at day 14. Data shown represent mean OD450 nm values (mean + SD) for each group of eight mice (**b**, **c**) and five mice (**d**, **e**). Serum IgG binding endpoint titers to (**c**) SARS-CoV-2 S1 + 2 and (**e**) SARS-CoV-2 RBD protein. Data representative of two independent experiments. Values depicted are mean +/− SD. *P* values determined by Mann–Whitney test.

Since candidate design, it has been observed that newly published SARS-CoV-2 Spike protein sequences match pGX9501 with >99.9% amino acid sequence identity (Supplementary Data 1). pGX9501 was therefore selected as the vaccine construct to advance to immunogenicity studies, due to the broader coverage it would likely provide compared with the outlier, pGX9503. pGX9501 was subsequently termed INO-4800. The immunogenicity of INO-4800 was evaluated in BALB/c mice, post-administration to the tibialis anterior muscle using the CELLECTRA® delivery device^16^. The reactivity of the sera from a group of mice immunized with INO-4800 was measured against a panel of SARS-CoV-2 and SARS-CoV antigens (Fig. 3a). Analysis revealed IgG binding against SARS-CoV-2 S protein antigens, with limited cross-reactivity to SARS-CoV S protein antigens, in the sera of INO-4800 immunized mice. We measured the serum IgG binding endpoint titers (EPTs) in mice immunized with pDNA against recombinant SARS-CoV-2 spike protein S1 + S2 regions (Fig. 3b, c) and recombinant SARS-CoV-2 spike protein receptor binding domain (RBD) (Fig. 3d, e). EPTs were observed in the sera of mice at day 14 after immunization with a single dose of INO-4800 (Fig. 3c, e).

**Fig. 4:**
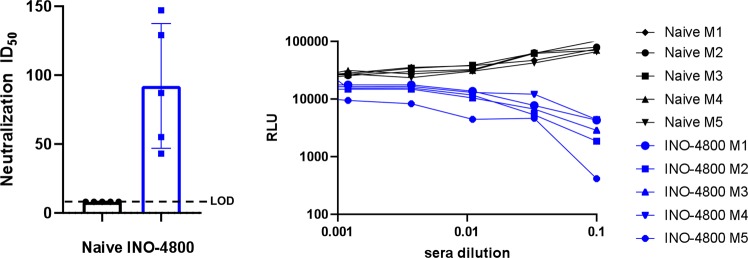
Neutralizing antibody responses after immunization of INO-4800. BALB/c mice (*n* of 5 per group) were immunized twice on days 0 and 14 with 10 µg of INO-4800. Sera was collected on day 7 post-second immunization and serial dilutions were incubated with a pseudovirus displaying the SARS-CoV-2 Spike and co-incubated with ACE2–293T cells. **a** Neutralization ID50 (mean ± SD) in naïve and INO-4800 immunized mice and **b** relative luminescence units (RLU) for sera from naive mice (green) and mice vaccinated with INO-4800 (red) as described in “Methods”.

We developed a neutralization assay with a pNL4–3.Luc.R-E-based pseudovirus displaying the SARS-CoV-2 Spike protein. Neutralization titers were detected by a reduction in relative luciferase units (RLU) compared with controls which had no decrease in RLU signal. BALB/c mice were immunized twice with INO-4800, on days 0 and 14, and sera was collected on day 7 post-second immunization. The pseudovirus was incubated with serial dilutions of mouse sera and the sera-virus mixture was added to 293T cells stably expressing the human ACE2 receptor (ACE2-293T) for 72 h. Neutralization ID50 average titers of 92.2 were observed in INO-4800 immunized mice (Fig. 4a, b). No reduction in RLU was observed for the control animals. Neutralizing titers were additionally measured against two wildtype SARS-CoV-2 virus strains by PRNT assay. Sera from INO-4800 immunized BALB/c mice neutralized both SARS-CoV-2/WH-09/human/2020 and SARS-CoV-2/Australia/VIC01/2020 virus strains with average ND50 titers of 97.5 and 128.1, respectively (Table 1). Live virus neutralizing titers were also evaluated in C57BL/6 mice following the same INO-4800 immunization regimen. Sera from INO-4800 immunized C57BL/6 mice neutralized wildtype SARS-CoV-2 virus with average ND50 titer of 340 (Table 1).

**Table 1:** Sera neutralizing activity after INO-4800 administration to mice and guinea pigs.

Model	Vaccine	*N*	Immunization regimen	Sample timepoint	Neutralization assay	Serum ND50 (reciprocal dilution)
BALB/c Mouse	pVAX	4	25 µg Days 0, 14	Day 21	SARS-CoV-2 (WH-09/human/2020)	<20, <20, <20, <20
	INO-4800	4	25 µg Days 0, 14	Day 21	SARS-CoV-2 (WH-09/human/2020)	30, 40, 80, 240
	pVAX	8	25 µg Days 0, 14	Day 21	SARS-CoV-2 (Australia/VIC01/2020)	<10, 12, 13, 15, 16, 17, 19, 24
	INO-4800	8	25 µg Days 0, 14	Day 21	SARS-CoV-2 (Australia/VIC01/2020)	27, 46, 91, 108, 130, 161, 221, 241
	pVAX	5	10 µg Days 0, 14	Day 21	SARS-CoV-2 Pseudovirus	8, 8, 8, 8, 8
	INO-4800	5	10 µg Days 0, 14	Day 21	SARS-CoV-2 Pseudovirus	43, 55, 87, 129, 147
C57BL/6 Mouse	pVAX	4	25 µg Days 0, 14	Day 21	SARS-CoV-2 (WH-09/human/2020)	<20, <20, <20, <20
	INO-4800	4	25 µg Days 0, 14	Day 21	SARS-CoV-2 (WH-09/human/2020)	240, 240, 240, 640
Guinea pig	pVAX	5	100 µg Days 0, 14, 28	Day 42	SARS-CoV-2 (Australia/VIC01/2020)	<10, 14, 20, 21, 25
	INO-4800	5	100 µg Days 0, 14, 28	Day 42	SARS-CoV-2 (Australia/VIC01/2020)	>320, >320, >320, >320, >320
	pVAX	5	100 µg Days 0, 14, 28	Day 35	SARS-CoV-2 Pseudovirus	<20, <20, <20, <20, <20
	INO-4800	5	100 µg Days 0, 14, 28	Day 35	SARS-CoV-2 Pseudovirus	527, 532, 579, 614, 616

### Humoral immune responses in guinea pigs

**Fig. 5:**
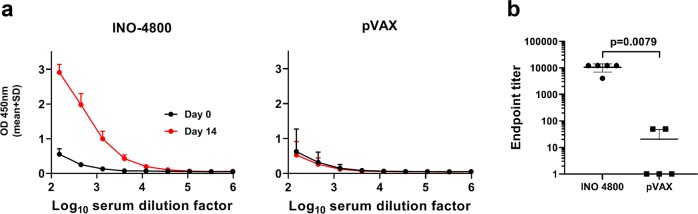
Humoral responses to SARS-CoV-2 in Hartley guinea pigs after a single dose of INO-4800. Hartley guinea pigs were immunized on Day 0 with 100 µg INO-4800 or pVAX-empty vector as described in the methods. **a** SARS-CoV-2 S protein antigen binding of IgG in serial serum dilutions at day 0 and 14. Data shown represent mean OD450 nm values (mean + SD) for the five guinea pigs. **b** Serum IgG binding titers (mean ± SD) to SARS-CoV-2 S protein at day 14. Values depicted are mean ± SD. *P* values determined by Mann–Whitney test.

We assessed the immunogenicity of INO-4800 in the Hartley guinea pig model, an established model for intradermal vaccine delivery^17,18^. One hundred micrograms of pDNA was administered by Mantoux injection to the skin and followed by CELLECTRA® delivery device on day 0 as described in the methods section. On day 14 anti-spike protein binding of serum antibodies was measured by ELISA. Immunization with INO-4800 revealed an immune response in respect to SARS-CoV-2 S1 + 2 protein binding IgG levels in the sera (Fig. 5a, b). The endpoint SARS-CoV-2 S protein binding titer at day 14 was 10,530 and 21 in guinea pigs treated with 100 µg INO-4800 or pVAX (control), respectively (Fig. 5b). We next evaluated antibody neutralizing activity following intradermal INO-4800 immunization in the guinea pig model. Guinea pigs were treated on days 0, 14, and 28 with pVAX or INO-4800, and sera samples were collected on days 35 or 42 to measure sera neutralizing activity against pseudovirus or wildtype virus, respectively. SARS-CoV-2 pseudovirus neutralizing activity with average ND50 titers of 573.5 was observed for the INO-4800 immunized guinea pigs (Table 1). Wildtype SARS-CoV-2 virus activity was also observed for the INO-4800 immunized guinea pigs with ND50 titers >320 by PRNT assay observed in all animals (Table 1).

### Inhibition of SARS-CoV-2 S protein binding to ACE2 receptor

**Fig. 6:**
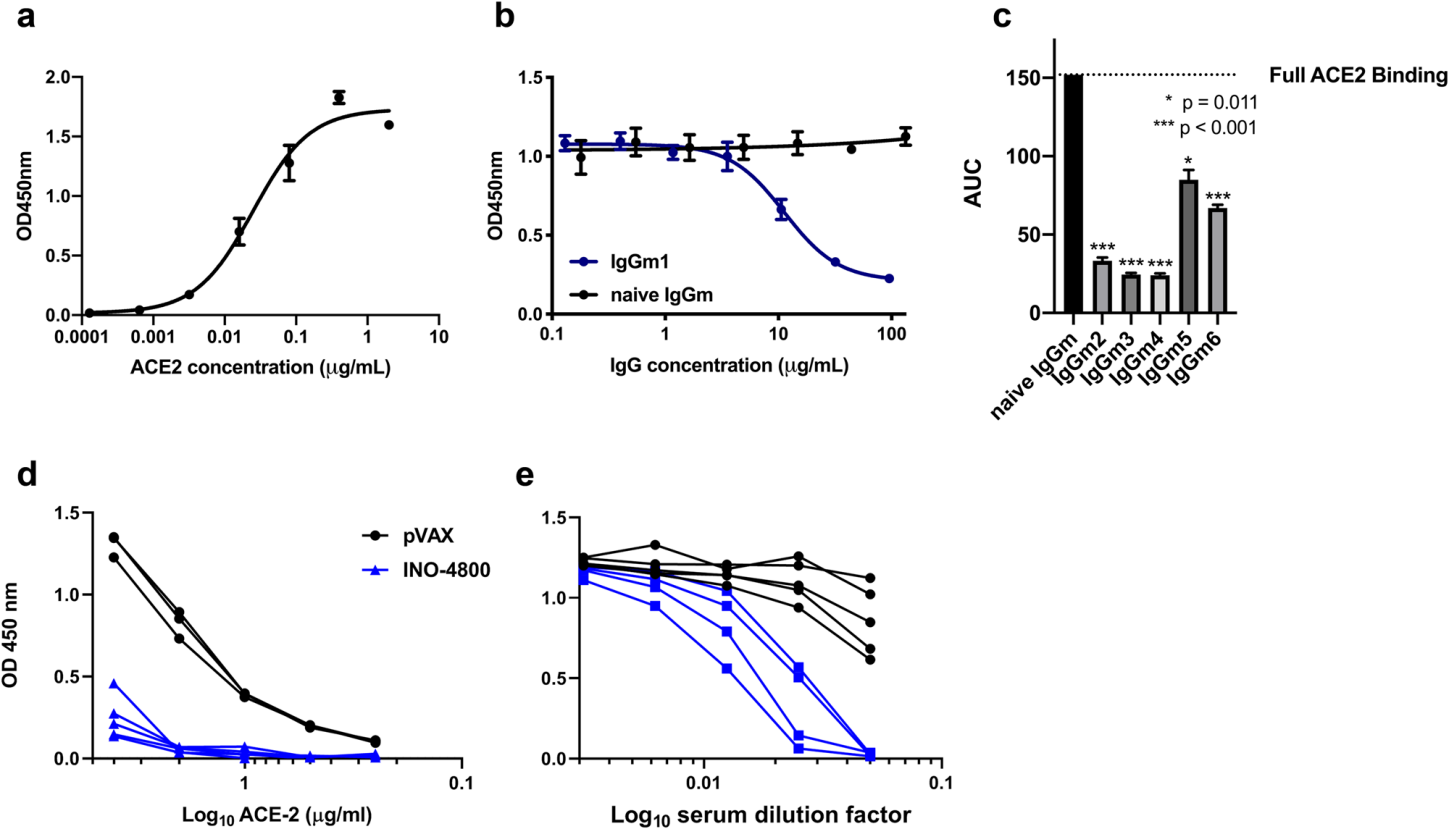
INO-4800 immunized mouse and guinea pig sera compete with ACE2 receptor for SARS-CoV-2 Spike protein binding. **a** Soluble ACE2 receptor binds to CoV-2 full-length spike with an EC_50_ of 0.025 µg/ml. **b** Purified serum IgG from BALB/c mice (*n* of 5 per group) after second immunization with INO-4800 yields significant competition against ACE2 receptor. Serum IgG samples from the animals were run in triplicate. **c** IgGs purified from *n* = 5 mice day 7 post second immunization with INO-4800 show significant competition against ACE2 receptor binding to SARS-CoV-2 S 1 + 2 protein. The soluble ACE2 concentration for the competition assay is ~0.1 µg ml^−1^. Bars represent the mean and standard deviation of AUC for curves displayed in Supplementary Fig. 1. **d** Hartley guinea pigs were immunized on Day 0 and 14 with 100 µg INO-4800 or pVAX-empty vector as described in the methods. Day 28 collected sera (diluted 1:20) was added SARS-CoV-2 coated wells prior to the addition of serial dilutions of ACE2 protein. Detection of ACE2 binding to SARS-CoV-2 S protein was measured. Sera collected from 5 INO-4800-treated and 3 pVAX-treated animals were used in this experiment. **e** Serial dilutions of guinea pig sera collected on day 21 were added to SARS-CoV-2 coated wells prior to the addition of ACE2 protein. Detection of ACE2 binding to SARS-CoV-2 S protein was measured. Sera collected from 4 INO-4800-treated and 5 pVAX-treated guinea pigs were used in this experiment.

The induction of antibodies capable of inhibiting Spike protein engagement of host receptor is considered relevant for SARS-CoV-2 vaccine development. We therefore examined the receptor inhibiting functionality of INO-4800-induced antibody responses. We recently developed an ELISA-based ACE2 inhibition assay as a surrogate for neutralization. The assay is similar in principle to other surrogate neutralization assays which have been validated for coronaviruses^19^. As a control in our assay, we show ACE2 can bind to SARS-CoV-2 Spike protein with an EC_50_ of 0.025 µg/ml (Fig. 6a). BALB/c mice were immunized on Days 0 and Day 14 with 10 µg of INO-4800, and serum IgG was purified on Day 21 post-immunization to ensure inhibition is antibody-mediated. We compared inhibition of the Spike-ACE2 interaction using serum IgG from a naïve mouse and from an INO-4800 vaccinated mouse (Fig. 6b). We repeated the receptor inhibition assay with a group of five immunized mice, and demonstrating that INO-4800-induced antibodies competed with ACE2 binding to the SARS-CoV-2 Spike protein (Fig. 6c and Supplementary Fig. 1). ACE2 binding inhibition was further evaluated in the guinea pig model. Sera collected from INO-4800 immunized guinea pigs inhibited binding of SARS-CoV-2 Spike protein over range of concentrations of ACE2 (0.25 µg/ml through 4 µg/ml) (Fig. 6d). Furthermore, serum dilution curves revealed sera collected from INO-4800 immunized guinea pigs blocked binding of ACE2 to SARS-CoV-2 in a dilution-dependent manner (Fig. 6e). Sera collected from pVAX-treated animals displayed negligible activity in the inhibition of ACE2 binding to the virus protein, the decrease in OD signal at the highest concentration of serum is considered a matrix effect in the assay. ACE2 is considered to be the primary receptor for SARS-CoV-2 cellular entry and blocking this interaction suggests INO-4800-induced antibodies believed important to prevent host infection.

In summary, humoral immunogenicity testing in both mice and guinea pigs revealed the COVID-19 vaccine candidate, INO-4800, was capable of eliciting functional blocking antibody responses to SARS-CoV-2 spike protein.

### Biodistribution of SARS-CoV-2 reactive IgG to the lung

**Fig. 7:**
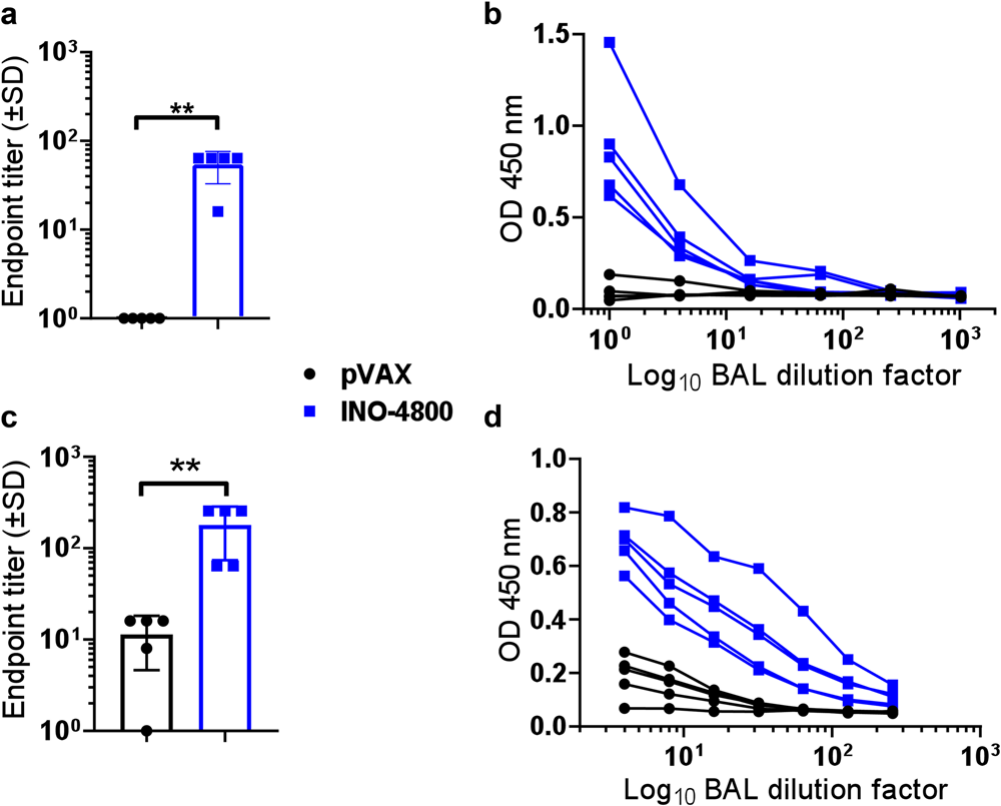
Detection of SARS-CoV-2 S protein-reactive antibodies in the BAL of INO-4800 immunized animals. BALB/c mice (n of 5 per group) were immunized on days 0 and 14 with INO-4800 or pVAX and BAL collected at day 21 (**a**, **b**). Hartley guinea pigs (*n* of 5 per group) were immunized on days 0, 14 and 21 with INO-4800 or pVAX and BAL collected at day 42 (**c**, **d**). Bronchoalveolar lavage fluid was assayed in duplicate for SARS-CoV-2 Spike protein-specific IgG antibodies by ELISA. Data are presented as endpoint titers (**a**, **c**), and BAL dilution curves with raw OD 450 nm values (**b**, **d**). **a**, **c** Bars represent the average of each group and error bars the standard deviation. **p < 0.01 by Mann–Whitney *U* test.

Lower respiratory disease (LRD) is associated with severe cases of COVID-19. The presence of antibodies at the lung mucosa targeting SARS-CoV-2 could potentially mediate protection against LRD. Therefore, we evaluated the presence of SARS-CoV-2 specific antibody in the lungs of immunized mice and guinea pigs. BALB/c mice and Hartley guinea pigs were immunized, on days 0 and 14 or 0, 14 and 28, respectively, with INO-4800 or pVAX control pDNA. Bronchoalveolar lavage (BAL) fluid was collected following sacrifice, and SARS-CoV-2 S protein ELISAs were performed. In both BALB/c and Hartley guinea pigs which received INO-4800 we measured a statistically significant increase in SARS-CoV-2 S protein binding IgG in BAL fluid compared with animals receiving pVAX control (Fig. 7a–d). Taken together, these data demonstrate the presence of anti-SARS-CoV-2 specific antibody in the lungs following immunization with INO-4800.

### Coronavirus cross-reactive cellular immune responses in mice

**Fig. 8:**
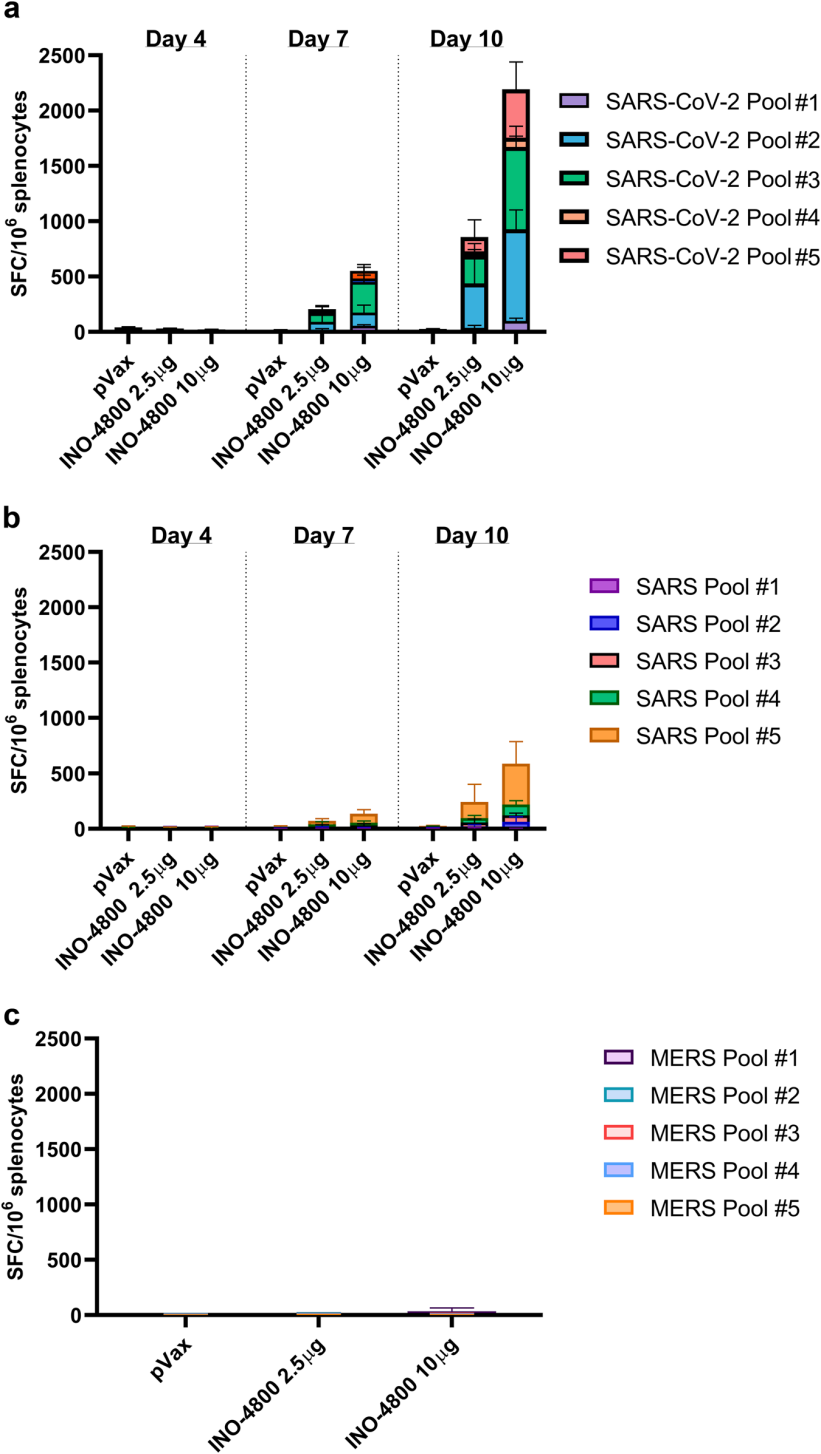
Induction of T cell responses in BALB/c mice post-administration of INO-4800. BALB/c mice (*n* = 5/group) were immunized with 2.5 or 10 µg INO-4800. T cell responses were analyzed in the animals on days 4, 7, 10 for plots a&b, and day 14 for plot c. T cell responses were measured by IFN-γ ELISpot in splenocytes stimulated for 20h with overlapping peptide pools spanning the SARS-CoV-2 (**a**), SARS-CoV (**b**), or MERS-CoV (**c**) Spike proteins. Bars represent the mean + SD. Data from individual mice is shown in Supplementary Data 2.

We assayed T cell responses against SARS-CoV-2, SARS-CoV, and MERS-CoV S antigens by IFN-γ ELISpot. Groups of BALB/c mice were sacrificed at days 4, 7, or 10 post-INO-4800 administration (2.5 or 10 μg of pDNA), splenocytes were harvested, and a single-cell suspension was stimulated for 20 h with pools of 15-mer overlapping peptides spanning the SARS-CoV-2, SARS-CoV, and MERS-CoV spike protein. Day 7 post-INO-4800 administration, we measured T cell responses of 205 and 552 SFU per 10^6^ splenocytes against SARS-CoV-2 for the 2.5 and 10 µg doses, respectively (Fig. 8a). Higher magnitude responses of 852 and 2193 SFU per 10^6^ splenocytes against SARS-CoV-2 were observed on Day 10 post-INO-4800 administration. Additionally, we assayed the cross-reactivity of the cellular response elicited by INO-4800 against SARS-CoV, observing detectable, albeit lower, T cell responses on both Day 7 (74 [2.5 µg dose] and 140 [10 µg dose] SFU per 10^6^ splenocytes) and Day 10 post-administration (242 [2.5 µg dose] and 588 [10 µg dose] SFU per 10^6^ splenocytes) (Fig. 8b). Interestingly, no cross-reactive T cell responses were observed against MERS-CoV peptides (Fig. 8c). Representative images of the IFN-γ ELISpot plates are provided in Supplementary Fig. 2. We proceeded to identify the T cell populations which were producing IFN-γ. Flow cytometric analysis on splenocytes harvested from BALB/c mice on Day 14 after a single INO-4800 immunization revealed the T cell compartment to contain 0.04% CD4+ and 0.32% CD8+ IFN-γ+ T cells after stimulation with SARS-CoV-2 antigens (Supplementary Fig. 3).

### BALB/c mouse SARS-CoV-2 epitope mapping

**Fig. 9:**
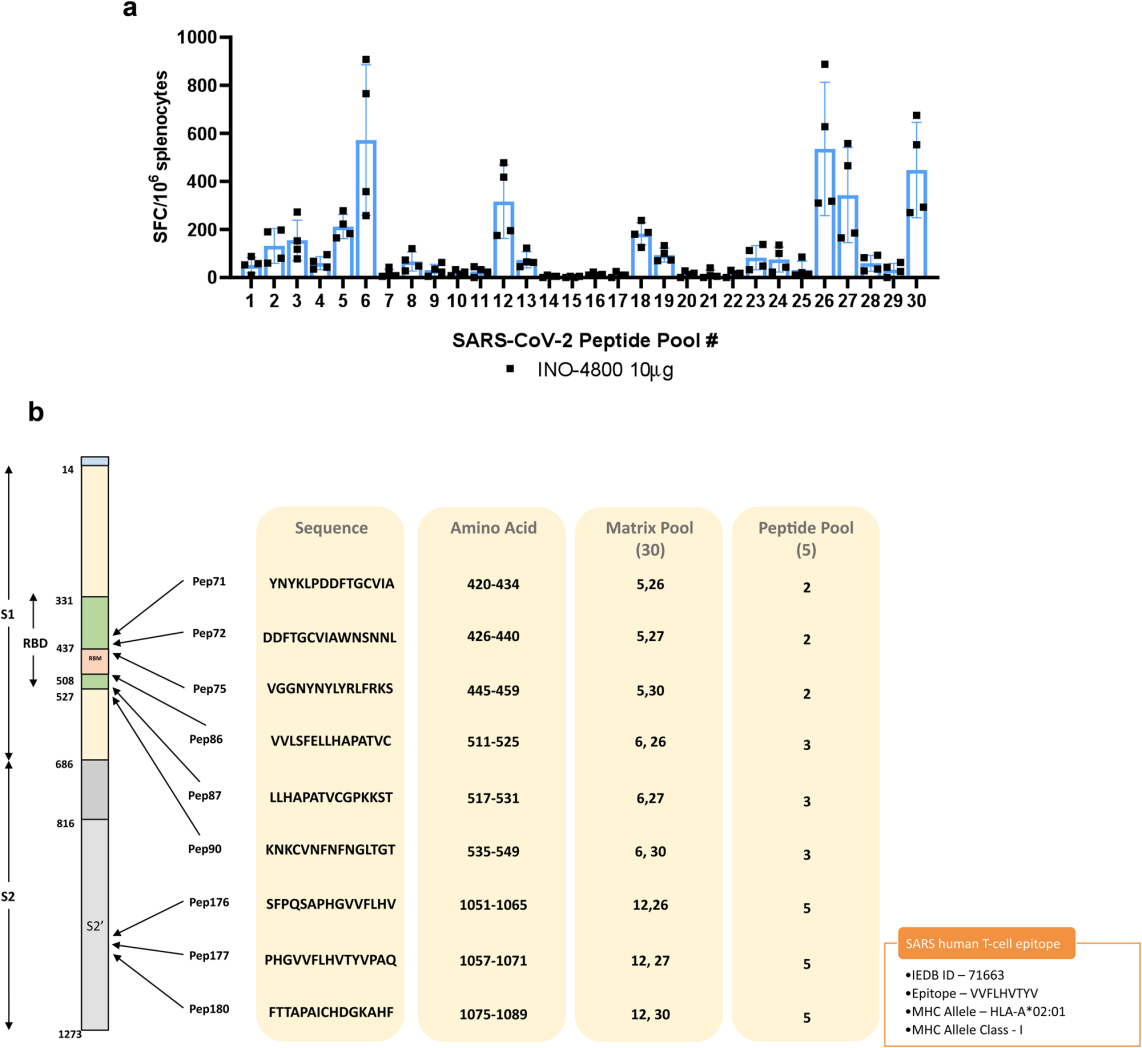
T cell epitope mapping after INO-4800 administration to BALB/c mice. Splenocytes were stimulated for 20 h with SARS-CoV-2 peptide matrix pools. **a** T cell responses following stimulation with matrix mapping SARS-CoV-2 peptide pools. Bars represent the mean + SD of five mice. **b** Map of the SARS-CoV-2 Spike protein and identification of immunodominant peptides in BALB/c mice. A known immunodominant SARS-CoV HLA-A2 epitope is included for comparison.

We performed epitope mapping on the splenocytes from BALB/c mice receiving the 10 µg INO-4800 dose. Thirty matrix mapping pools were used to stimulate splenocytes for 20 h and immunodominant responses were detected in multiple peptide pools (Fig. 9a). The responses were deconvoluted to identify several epitopes (H2-K^d^) clustering in the receptor binding domain and in the S2 domain (Fig. 9b). Interestingly, one SARS-CoV-2 H2-K^d^ epitope, PHGVVFLHV, was observed to be overlapping and adjacent to the SARS-CoV human HLA-A2 restricted epitope VVFLHVTYV^20^.

In summary, T cell responses against SARS-CoV-2 S protein epitopes were detected in mice immunized with INO-4800.

## Discussion

The novel coronavirus, SARS-CoV-2, and associated COVID-19 disease has become a global pandemic with a significant morbidity and mortality toll. Currently, there are no COVID-19 vaccines available, and global dissemination of SARS-CoV-2 may continue until there is a high level of herd immunity within the human population. Here we have described the preclinical development of a synthetic DNA-based COVID-19 vaccine, INO-4800 to combat this emerging infectious disease. Synthetic DNA vaccine design and synthesis was immediately initiated upon public release of the SARS-CoV-2 genome sequences on 11 January 2020. Our data support the expression and immunogenicity of the INO-4800 synthetic DNA vaccine candidate in multiple animal models. Humoral and T cell responses were observed in mice. In guinea pigs we employed clinical delivery parameters and observed SARS-CoV-2 S protein binding antibody titers and blocking of ACE2/SARS-CoV-2 S protein interaction in serum samples from INO-4800-treated animals. nAbs were also measured in both species.

Halting a rapidly emerging infectious disease requires an orchestrated response from the global health community and requires improved strategies to accelerate vaccine development. In response to the 2019/2020 coronavirus outbreak we employed a synthetic DNA medicine platform. The design and manufacture of this synthetic DNA vaccine represents a plug and play process in which we insert the target antigen sequence into a highly characterized and clinically tested plasmid vector backbone (pGX0001). The construct design and engineering parameters have been optimized for in vivo gene expression, and previously applied to MERS, EBOV, Zika, and Lassa DNA vaccine constructs which are all undergoing clinical testing^7,9,11,21,22^.

Based upon our previous experience developing a vaccine against MERS coronavirus, and previous published studies of SARS vaccines, SARS-CoV-2 S protein was chosen as the antigen target. The SARS-CoV-2 S protein is a class I membrane fusion protein, which the major envelope protein on the surface of coronaviruses. Initial studies have already been performed which indicate SARS-CoV-2 interaction with its host receptor (ACE2) can be blocked by antibodies^23^. In vivo immunogenicity studies in both mouse and guinea pig models revealed levels of S protein-reactive IgG in the serum of INO-4800 immunized animals. In addition to full-length S1 + S2 and S1, INO-4800 immunization induced RBD binding antibodies (Fig. 3), a domain known to be a target for nAbs from SARS-CoV convalescent patients^24,25^. We further demonstrate the functionality of these antibodies through neutralization of SARS-CoV-2 wild-type virus and pseudovirus (Table 1), and competitive inhibition of SARS-CoV-2 spike protein binding to the ACE2 receptor in the presence of sera from INO-4800 immunized animals (Fig. 6). Importantly, anti-SARS-CoV-2 binding antibodies were detected in lung washes of INO-4800-immunized mice and guinea pigs (Fig. 7). The presence of these antibodies in the lungs has the potential to protect against infection of these tissues and prevent LRD, which is associated with the severe cases of COVID-19. In addition to humoral responses, cellular immune responses have been shown to be associated with more favorable recovery in MERS-CoV infection^26^, and are likely to be important against SARS-CoV infection^27^. Here, we showed the induction of T cell responses against SARS-CoV-2 as early as day 7 post-vaccine delivery. Rapid cellular responses have the potential to lower viral load and could potentially reduce the spread of SARS-CoV-2 and the associated COVID-19 illness.

We believe synthetic DNA medicine platform has several synergistic characteristics which position it well to respond to disease outbreaks, such as COVID-19. As mentioned previously, the ability to design and immediately synthesize candidate vaccine constructs means that in vitro and in vivo testing can potentially begin within days of receiving the viral sequence. The DNA plasmid manufacture process allows for scalable manufacture of drug product, which has the potential to circumvent the complexities of conventional vaccine production in eggs or cell culture. Additionally, we have published on the stability profile afforded to these products through the use of our optimized DNA formulation^7^. The stability characteristics mean that our DNA drug product is non-frozen and can be stored for 4.5+ years at 2–8 °C, room temperature (RT) for 1 year and 1 month at 37 °C, while maintaining potency at temperatures upwards of 60 °C. In the context of a pandemic outbreak, the stability profile of a vaccine plays directly to its ability to be deployed and stockpiled in an efficient and executable manner. In this study, we observed seroconversion after a single intradermal administration of the INO-4800 in guinea pigs (Fig. 6). Whether a single immunization will be sufficient in humans will be investigated in clinical trials.

Although vaccine-induced immunopathology has been raised as a potential concern for SARS and MERS vaccine candidates, and possibly for SARS-CoV-2 vaccines, these concerns are likely vaccine-platform dependent and, to-date, no evidence of immune pathogenesis has been reported for MERS DNA vaccines in mice or non-human primate models^10^ or SARS DNA vaccines in mice^8^. Lung immunopathology characterized by Th2-related eosinophilia has been reported for whole inactivated virus (IV), recombinant protein, peptide, and/or recombinant viral vector vaccines following SARS-CoV challenge^28–32^, and more recently in a MERS-CoV challenge model^33^. However, in the majority of studies protective efficacy without lung immunopathology has been reported for SARS-CoV and MERS-CoV vaccines^8,10,34–40^. It is important to note the majority of studies demonstrating CoV vaccine-induced immunopathology utilized the BALB/c mouse, a model known to preferentially develop Th2-type responses. The DNA vaccine platform induces Th1-type immune responses and has demonstrated efficacy without immunopathology in models of respiratory infection, including SARS-CoV^8^, MERS-CoV^10^, and RSV^41^. SARS-CoV-2 animal challenge studies will assess INO-4800-mediated protection against disease, and vaccine-enhanced disease.

Here, we report functional neutralization of INO-4800 immune sera using a SARS-CoV-2 pseudovirus assay (Fig. 4, Table 1), and PRNT assay against two wild-type SARS-CoV-2 strains (Table 1). As well, we show that INO-4800 induced antibodies block SARS-CoV-2 Spike binding to the host receptor ACE2, using a surrogate neutralization assay (Fig. 6). This study highlights the immunogenicity of INO-4800, and further animal studies will test protection against infection.

In summary, these initial results describing the immunogenicity of COVID-19 vaccine candidate, INO-4800 are promising, and it is particularly encouraging to measure functional antibodies and T cell responses in multiple animal models. This study supports the further evaluation of INO-4800 as a vaccine candidate for COVID-19.

## Methods

### Cell lines

HEK-293T (ATCC® CRL-3216™) and African Green monkey kidney COS-7 (ATCC® CRL-1651™) cell lines were obtained from ATCC (Old Town Manassas, VA). All cell lines were maintained in DMEM supplemented with 10% fetal bovine serum (FBS) and penicillin-streptomycin.

In vitro RNA expression (qRT-PCR) In vitro mRNA expression of the plasmid was demonstrated by transfection of COS-7 with serially diluted plasmids followed by analysis of the total RNA extracted from the cells using reverse transcription and PCR. Transfections of four concentrations of the plasmid were performed using FuGENE® 6 transfection reagent (Promega) which resulted in final masses ranging between 80 and 10 ng per well. The transfections were performed in duplicate. Following 18 to 26 h of incubation the cells were lysed with RLT Buffer (Qiagen). Total RNA was isolated from each well using the Qiagen RNeasy kit following the kit instructions. The resulting RNA concentration was determined by OD_260/280_ and samples of the RNA were diluted to 10 ng per µL. One hundred nanograms of RNA was then converted to cDNA using the High Capacity cDNA Reverse Transcription (RevT) kit (Applied Biosystems) following the kit instructions. RevT reactions containing RNA but no reverse transcriptase (minus RT) were included as controls for plasmid DNA or cellular genomic DNA sample contamination. Eight microliters of sample cDNA were then subjected to PCR using primers and probes that are specific to the target sequence (pGX9501 Forward – CAGGACAAGAACACACAGGAA; pGX9501 Reverse – CAGGCAGGATTTGGGAGAAA; pGX9501 Probe – ACCCATCAAGGACTTTGGAGG; and pGX9503 Forward – AGGACAAGAACACACAGGAAG; pGX9503 Reverse – CAGGATCTGGGAGAAGTTGAAG; pGX9503 Probe – ACACCACCCATCAAGGACTTTGGA). In a separate reaction, the same quantity of sample cDNA was subjected to PCR using primers and a probe designed (β-actin Forward – GTGACGTGGACATCCGTAAA; β-actin Reverse – CAGGGCAGTAATCTCCTTCTG; β-actin Probe – TACCCTGGCATTGCTGACAGGATG) for COS-7 cell line β-actin sequences. The primers and probes were synthesized by Integrated DNA Technologies, Inc. and the probes were labeled with 56-FAM and Black Hole Quencher 1. The reaction used ABI Fast Advance 2×(Cat. No. 4444557), with final forward and reverse primer concentrations of 1 µM and probe concentrations of 0.3 µM. Using a QuantStudio 7 Flex Real Time PCR Studio System (Applied Biosystems), samples were first subjected to a hold of 1 min at 95 °C and then 40 cycles of PCR with each cycle consisting of 1 s at 95 °C and 20 s at 60 °C. Following PCR, the amplifications results were analyzed as follows. The negative transfection controls, the minus RevT controls, and the NTC were scrutinized for each of their respective indications. The threshold cycle (C_T_) of each transfection concentration for the INO-4800 COVID-19 target mRNA and for the β-actin mRNA was generated from the QuantStudio software using an automatic threshold setting. The plasmid was considered to be active for mRNA expression if the expression in any of the plasmid transfected wells compared with the negative transfection controls were greater than 5 C_T_.

### In vitro protein expression (Western blot)

Human embryonic kidney cells, 293T were cultured and transfected as described previously^42^. 293T cells were transfected with pDNA using TurboFectin8.0 (OriGene) transfection reagent following the manufacturer’s protocol. Forty-eight hours later cell lysates were harvested using modified RIPA cell lysis buffer. Proteins were separated on a 4–12% BIS-TRIS gel (ThermoFisher Scientific), then following transfer, blots were incubated with an anti-SARS-CoV spike protein polyclonal antibody (Novus Biologicals) then visualized with horseradish peroxidase (HRP)-conjugated anti-mouse IgG (GE Amersham).

### Immunofluorescence of transfected 293T cells

For in vitro staining of Spike protein expression 293T cells were cultured on 4-well glass slides (Lab-Tek) and transfected with 3 µg per well of pDNA using TurboFectin8.0 (OriGene) transfection reagent following the manufacturer’s protocol. Cells were fixed 48 h after transfection with 10% Neutral-buffered Formalin (BBC Biochemical, Washington State) for 10 min at RT and then washed with PBS. Before staining, chamber slides were blocked with 0.3% (v/v) Triton-X (Sigma), 2% (v/v) donkey serum in PBS for 1 h at RT. Cells were stained with a rabbit anti-SARS-CoV spike protein polyclonal antibody (Novus Biologicals) diluted in 1% (w/v) BSA (Sigma), 2% (v/v) donkey serum, 0.3% (v/v) Triton-X (Sigma) and 0.025% (v/v) 1 g ml^−1^ Sodium Azide (Sigma) in PBS for 2 h at RT. Slides were washed three times for 5 min in PBS and then stained with donkey anti-rabbit IgG AF488 (lifetechnologies, A21206) for 1 h at RT. Slides were washed again and mounted and covered with DAPI-Fluoromount (SouthernBiotech).

### Animals

Female, 6-week-old C57/BL6 and BALB/c mice were purchased from Charles River Laboratories (Malvern, PA) and The Jackson Laboratory (Bar Harbor, ME). Female, 8-week-old Hartley guinea pigs were purchased from Elm Hill Labs (Chelmsford, MA). All animals were housed in the animal facility at The Wistar Institute Animal Facility or Acculab Life Sciences (San Diego, CA). All animal testing and research complied with all relevant ethical regulations and studies received ethical approval by the Wistar Institute or Acculab Institutional Animal Care and Use Committees (IACUC). For mouse studies, on day 0 doses of 2.5, 10 or 25 µg pDNA were administered to the tibialis anterior (TA) muscle by needle injection followed by CELLECTRA® in vivo electroporation (EP). The CELLECTRA® EP delivery consists of two sets of pulses with 0.2 Amp constant current. Second pulse sets is delayed 3 s. Within each set there are two 52 ms pulses with a 198 ms delay between the pulses. On days 0 and 14 blood was collected. Parallel groups of mice were serially sacrificed on days 4, 7, and 10 post-immunization for analysis of cellular immune responses. For guinea pig studies, on day 0, 100 µg pDNA was administered to the skin by Mantoux injection followed by CELLECTRA® in vivo EP.

### Antigen binding ELISA

ELISAs were performed to determine sera antibody binding titers. Nunc ELISA plates were coated with 1 µg ml^−1^ recombinant protein antigens in Dulbecco’s phosphate-buffered saline (DPBS) overnight at 4 °C. Plates were washed three times then blocked with 3% bovine serum albumin (BSA) in DPBS with 0.05% Tween 20 for 2 h at 37 °C. Plates were then washed and incubated with serial dilutions of mouse or guinea pig sera and incubated for 2 h at 37 °C. Plates were again washed and then incubated with 1:10,000 dilution of horse radish peroxidase (HRP) conjugated anti-guinea pig IgG secondary antibody (Sigma-Aldrich, cat. A7289) or (HRP) conjugated anti-mouse IgG secondary antibody (Sigma-Aldrich) and incubated for 1 h at RT. After final wash plates were developed using SureBlue^TM^ TMB 1-Component Peroxidase Substrate (KPL, cat. 52-00-03) and the reaction stopped with TMB Stop Solution (KPL, cat. 50-85-06). Plates were read at 450 nm wavelength within 30 min using a Synergy HTX (BioTek Instruments, Highland Park, VT). Binding antibody EPTs were calculated as previously described^43^. Binding antigens tested included, SARS-CoV-2 antigens: S1 spike protein (Sino Biological 40591-V08H), S1 + S2 ECD spike protein (Sino Biological 40589-V08B1), RBD (University of Texas, at Austin (McLellan Lab.)); SARS-COV antigens: Spike S1 protein (Sino Biological 40150-V08B1), S (1-1190) (Immune Tech IT-002-001P) and Spike C-terminal (Meridian Life Science R18572).

### ACE2 competition ELISA

For mouse studies, ELISAs were performed to determine sera IgG antibody competition against human ACE2 with a human Fc tag. Nunc ELISA plates were coated with 1 µg mL^−1^ rabbit anti-His6X in 1× PBS for 4–6 h at RT and washed four times with washing buffer (1× PBS and 0.05% Tween 20). Plates were blocked overnight at 4 °C with blocking buffer (1× PBS, 0.05% Tween 20, 5% evaporated milk and 1% FBS). Plates were washed four times with washing buffer then incubated with full length (S1 + S2) spike protein containing a C-terminal His tag (Sino Biologics, cat. 40589-V08B1) at 10 µg mL^−1^ for 1 h at RT. Plates were washed and then serial dilutions of purified mouse IgG mixed with 0.1 µg mL^−1^ recombinant human ACE2 with a human Fc tag (ACE2-IgHu) were incubated for 1–2 h at RT. Plates were again washed and then incubated with 1:10,000 dilution of HRP conjugated anti-human IgG secondary antibody (Bethyl, cat. A80-304P) and incubated for 1 h at RT. After final wash plates were developed using 1-Step Ultra TMB-ELISA Substrate (Thermo, cat. 34029) and the reaction stopped with 1 M Sulfuric Acid. Plates were read at 450 nm wavelength within 30 min using a SpectraMax Plus 384 Microplate Reader (Molecular Devices, Sunnyvale, CA). Competition curves were plotted and the area under the curve (AUC) was calculated using Prism 8 analysis software with multiple *t*-tests to determine statistical significance.

For guinea pig studies, 96-well half area assay plates (Costar) were coated with 25 µl per well of 5 µg mL^−1^ of SARS-CoV-2 spike S1 + S2 protein (Sino Biological) diluted in 1× DPBS (Thermofisher) overnight at 4 °C. Plates were washed with 1× PBS buffer with 0.05% TWEEN (Sigma). Hundred microliters per well of 3% (w/v) BSA (Sigma) in 1× PBS with 0.05% TWEEN were added and incubated for 1 h at 37 °C. Serum samples were diluted 1:20 in 1% (w/v) BSA in 1× PBS with 0.05% TWEEN. After washing the assay plate, 25 µl/well of diluted serum was added and incubated 1 h at 37 °C. Human recombinant ACE2-Fc-tag (Sinobiological) was added directly to the diluted serum, followed by 1 h of incubation at 37 °C. Plates were washed and 25 µl per well of 1:10,000 diluted goat anti-hu Fc fragment antibody HRP (Bethyl, A80-304P) was added to the assay plate. Plates were incubated 1 h at RT. For development the SureBlue/TMB Stop Solution (KPL, MD) was used and O.D. was recorded at 450 nm.

### SARS-CoV-2 Pseudovirus neutralization assay

SARS-CoV-2 pseudotyped viruses were produced using HEK293T cells transfected with GeneJammer (Agilent) using IgE-SARS-CoV-2 S plasmid (Genscript) and pNL4-3.Luc.R-E- plasmid (NIH AIDS reagent) at a 1:1 ratio. Forty-eight hours post transfection, transfection supernatant was collected, enriched with FBS to 12% final volume, steri-filtered (Millipore Sigma), and aliquoted for storage at −80 °C. SARS-CoV-2 pseudotyped viruses were titered and yielding >50 times the relative luminescence units (RLU) to cells alone after 72 h of infection. Mouse sera from INO-4800 vaccinated and naive groups were heat inactivated for 15 min at 56 °C and serially diluted threefold starting at a 1:10 dilution for assay. Sera were incubated with a fixed amount of SARS-Cov-2 pseudotyped virus for 90 min. HEK293T cells stably expressing ACE2 were added after 90 min and allowed to incubate in standard incubator (37% humidity, 5% CO_2_) for 72 h. Post infection, cells were lysed using britelite plus luminescence reporter gene assay system (Perkin Elmer Catalog no. 6066769) and RLU were measured using the Biotek plate reader. Neutralization titers (ID_50_) were calculated as the serum dilution at which RLU were reduced by 50% compared with RLU in virus control wells after subtraction of background RLU in cell control wells.

### SARS-CoV-2 wildtype virus neutralization assays

SARS-CoV-2/Australia/VIC01/2020 isolate neutralization assays were performed at Public Health England (Porton Down, UK). Neutralizing virus titers were measured in serum samples that had been heat-inactivated at 56 °C for 30 min. SARS-CoV-2 (Australia/VIC01/2020 isolate^44^) was diluted to a concentration of 933 pfu ml^−1^ and mixed 50:50 in 1% FCS/MEM containing 25 mM HEPES buffer with doubling serum dilutions from 1:10 to 1:320 in a 96-well V-bottomed plate. The plate was incubated at 37 °C in a humidified box for 1 h before the virus was transferred into the wells of a twice DPBS-washed 24-well plate that had been seeded the previous day at 1.5 × 10^5^ Vero E6 cells per well in 10% FCS/MEM. Virus was allowed to adsorb at 37 °C for a further hour, and overlaid with plaque assay overlay media (1× MEM/1.5% CMC/4% FCS final). After 5 days incubation at 37 °C in a humidified box, the plates were fixed, stained and plaques counted. Median neutralizing titers (ND50) were determined using the Spearman–Karber formula relative to virus only control wells.

SARS-CoV-2/WH-09/human/2020 isolate neutralization assays were performed at the Institute of Laboratory Animal Science, Chinese Academy of Medical Sciences (CAMS) approved by the National Health Commission of the People’s Republic of China. Seed SARS-CoV-2 (SARS-CoV-2/WH-09/human/2020) stocks and virus isolation studies were performed in Vero E6 cells, which are maintained in Dulbecco’s modified Eagle’s medium (DMEM, Invitrogen, Carlsbad, USA) supplemented with 10% fetal bovine serum (FBS), 100 IU ml^−1^ penicillin, and 100 µg ml^−1^ streptomycin, and incubated at 36.5 °C, 5% CO_2_. Virus titer were determined using a standard 50% tissue culture infection dose (TCID50) assay. Serum samples collected from immunized animals were inactivated at 56 °C for 30 min and serially diluted with cell culture medium in two-fold steps. The diluted samples were mixed with a virus suspension of 100 TCID_50_ in 96-well plates at a ratio of 1:1, followed by 2 h incubation at 36.5 °C in a 5% CO_2_ incubator. 1–2 × 10^4^ Vero cells were then added to the serum-virus mixture, and the plates were incubated for 3–5 days at 36.5 °C in a 5% CO_2_ incubator. Cytopathic effect (CPE) of each well was recorded under microscopes, and the neutralizing titer was calculated by the dilution number of 50% protective condition.

### BAL collection

BAL fluid was collected by washing the lungs of euthanized and exsanguinated mice with 700–1000 μl of ice-cold PBS containing 100 μm EDTA, 0.05% sodium azide, 0.05% Tween-20, and 1× protease inhibitor (Pierce) (mucosal prep solutions (MPS) with a blunt-ended needle. Guinea pig lungs were washed with 20 ml of MPS via 16 G catheter inserted into the trachea. Collected BAL fluid was stored at −20C until the time of assay.

### IFN-γ ELISpot

Spleens from mice were collected individually in RPMI1640 media supplemented with 10% FBS (R10) and penicillin/streptomycin and processed into single cell suspensions. Cell pellets were re-suspended in 5 mL of ACK lysis buffer (Life Technologies, Carlsbad, CA) for 5 min RT, and PBS was then added to stop the reaction. The samples were again centrifuged at 1500 × *g* for 10 min, cell pellets re-suspended in R10, and then passed through a 45 µm nylon filter before use in ELISpot assay. ELISpot assays were performed using the Mouse IFN-*γ* ELISpot^PLUS^ plates (MABTECH). 96-well ELISpot plates pre-coated with capture antibody were blocked with R10 medium overnight at 4 °C. 200,000 mouse splenocytes were plated into each well and stimulated for 20 h with pools of 15-mer peptides overlapping by nine amino acid from the SARS-CoV-2, SARS-CoV, or MERS-CoV Spike proteins (five peptide pools per protein). Additionally, matrix mapping was performed using peptide pools in a matrix designed to identify immunodominant responses. Cells were stimulated with a final concentration of 5 μL of each peptide per well in RPMI + 10% FBS (R10). The spots were developed based on manufacturer’s instructions. R10 and cell stimulation cocktails (Invitrogen) were used for negative and positive controls, respectively. Spots were scanned and quantified by ImmunoSpot CTL reader. Spot-forming unit (SFU) per million cells was calculated by subtracting the negative control wells.

### Flow cytometry

Intracellular cytokine staining was performed on splenocytes harvested from BALB/c and C57BL/6 mice stimulated with the overlapping peptides spanning the SARS-CoV-2 S protein for 6 h at 37 °C, 5% CO_2_. Cells were stained with the following antibodies from BD Biosciences, unless stated, with the dilutions stated in parentheses: FITC anti-mouse CD107a (1:100), PerCP-Cy5.5 anti-mouse CD4 (1:100), APC anti-mouse CD8a (1:100), ViViD Dye (1–40) (LIVE/DEAD® Fixable Violet Dead Cell Stain kit; Invitrogen, L34955), APC-Cy7 anti-mouse CD3e (1:100), and BV605 anti-mouse IFN-γ (1:75) (eBiosciences). Phorbol Myristate Acetate (PMA) were used as a positive control, and complete medium only as the negative control. Cells were washed, fixed and, cell events were acquired using an FACS CANTO (BD Biosciences), followed by FlowJo software (FlowJo LLC, Ashland, OR) analysis.

### Structural modeling

The structural models for SARS-CoV and MERS-CoV were constructed from PDB IDs 6ACC and 5×59 in order to assemble a prefusion model with all three RBDs in the down conformation. The SARS-CoV-2 structural model was built by using SARS-CoV structure (PDB ID:6ACC) as a template. Rosetta remodel simulations were employed to make the appropriate amino acid mutations and to build de novo models for SARS-CoV-2 loops not structurally defined in the SARS-CoV structure^45^. Amino acid positions neighboring the loops were allowed to change backbone conformation to accommodate the new loops. The structural figures were made using PyMOL.

### Statistics

All statistical analyses were performed using GraphPad Prism 7 or 8 software (La Jolla, CA). These data were considered significant if *p* < 0.05. The lines in all graphs represent the mean value and error bars represent the standard deviation. No samples or animals were excluded from the analysis. Randomization was not performed for the animal studies. Samples and animals were not blinded before performing each experiment.

### Reporting summary

Further information on research design is available in the Nature Research Reporting Summary linked to this article.

## Supplementary information

Supplementary Information

Reporting Summary

Description of Additional Supplementary Files

Supplementary Data 1

Supplementary Data 2

## Data Availability

The authors declare that all data supporting the findings of the study are available in this article and its Supplementary Information files, or from the corresponding author The source data underlying Figs. 2b–d, 3a–e, 4a, b, 5a, b, 6a–e, 7a–d, 8a–c, 9a and Supplementary Figs. 1, 3b are provided as a Source Data file.

## Source data

Source data

## References

[1] Zhu V (2020). A Novel Coronavirus from Patients with Pneumonia in China, 2019. N. Engl. J. Med..

[2] Wu F (2020). A new coronavirus associated with human respiratory disease in China. Nature.

[3] Huang C (2020). Clinical features of patients infected with 2019 novel coronavirus in Wuhan, China. Lancet.

[4] GISAID. Coronavirus COVID-19 Global Cases by Johns Hopkins CSSE. GISAID. https://www.gisaid.org/epiflu-applications/global-cases-covid-19/ (2020).

[5] Voytko, L. Chinese healthcare workers are facing a surgical mask shortage amid coronavirus panic. https://www.forbes.com/sites/lisettevoytko/2020/02/07/chinese-healthcare-workers-are-now-facing-a-surgical-mask-shortage-amid-coronavirus-panic/#f1d7d4a72e78 (2020).

[6] Hulkower RL, Casanova LM, Rutala WA, Weber DJ, Sobsey MD (2011). Inactivation of surrogate coronaviruses on hard surfaces by health care germicides. Am. J. Infect. Control.

[7] Tebas P (2019). Intradermal SynCon(R) Ebola GP DNA vaccine is temperature stable and safely demonstrates cellular and humoral immunogenicity advantages in healthy volunteers. J. Infect. Dis..

[8] Yang ZY (2004). A DNA vaccine induces SARS coronavirus neutralization and protective immunity in mice. Nature.

[9] Modjarrad K (2019). Safety and immunogenicity of an anti-Middle East respiratory syndrome coronavirus DNA vaccine: a phase 1, open-label, single-arm, dose-escalation trial. Lancet Infect. Dis..

[10] Muthumani K (2015). A synthetic consensus anti-spike protein DNA vaccine induces protective immunity against Middle East respiratory syndrome coronavirus in nonhuman primates. Sci. Transl. Med..

[11] Tebas, P. et al. Safety and Immunogenicity of an Anti-Zika Virus DNA Vaccine - Preliminary Report. *N. Engl. J. Med.*10.1056/NEJMoa1708120 (2017). 10.1056/NEJMoa1708120 PMC6824915 34525286

[12] Wrapp, D. et al. Cryo-EM structure of the 2019-nCoV spike in the prefusion conformation. *Science*, eabb2507 (2020). 10.1126/science.abb2507 PMC7164637 32075877

[13] Kirchdoerfer RN (2018). Stabilized coronavirus spikes are resistant to conformational changes induced by receptor recognition or proteolysis. Sci. Rep..

[14] Wang, L. et al. Importance of neutralizing monoclonal antibodies targeting multiple antigenic sites on the Middle East respiratory syndrome coronavirus spike glycoprotein to avoid neutralization escape. *J. Virol.* **92**, e02002-17 (2018). 10.1128/JVI.02002-17 PMC5923077 29514901

[15] Tian X (2020). Potent binding of 2019 novel coronavirus spike protein by a SARS coronavirus-specific human monoclonal antibody. Emerg. Microbes Infect..

[16] Sardesai NY, Weiner DB (2011). Electroporation delivery of DNA vaccines: prospects for success. Curr. Opin. Immunol..

[17] Carter D (2018). The adjuvant GLA-AF enhances human intradermal vaccine responses. Sci. Adv..

[18] Schultheis K (2017). Characterization of guinea pig T cell responses elicited after EP-assisted delivery of DNA vaccines to the skin. Vaccine.

[19] Rosen O (2019). A high-throughput inhibition assay to study MERS-CoV antibody interactions using image cytometry. J. Virol. Methods.

[20] Ahmed S, Quadeer AA, McKay MR (2020). Preliminary identification of potential vaccine targets for the COVID-19 coronavirus (SARS-CoV-2) based on SARS-CoV immunological studies. Viruses.

[21] Patel A (2019). Protective efficacy and long-term immunogenicity in cynomolgus macaques by Ebola virus glycoprotein synthetic DNA vaccines. J. Infect. Dis..

[22] Jiang J (2019). Immunogenicity of a protective intradermal DNA vaccine against lassa virus in cynomolgus macaques. Hum. Vaccin Immunother..

[23] Zhou P (2020). A pneumonia outbreak associated with a new coronavirus of probable bat origin. Nature.

[24] Zhu Z (2007). Potent cross-reactive neutralization of SARS coronavirus isolates by human monoclonal antibodies. Proc. Natl Acad. Sci. USA.

[25] He Y (2005). Identification of a critical neutralization determinant of severe acute respiratory syndrome (SARS)-associated coronavirus: importance for designing SARS vaccines. Virology.

[26] Zhao Jingxian, Alshukairi Abeer N., Baharoon Salim A., Ahmed Waleed A., Bokhari Ahmad A., Nehdi Atef M., Layqah Laila A., Alghamdi Mohammed G., Al Gethamy Manal M., Dada Ashraf M., Khalid Imran, Boujelal Mohamad, Al Johani Sameera M., Vogel Leatrice, Subbarao Kanta, Mangalam Ashutosh, Wu Chaorong, Ten Eyck Patrick, Perlman Stanley, Zhao Jincun (2017). Recovery from the Middle East respiratory syndrome is associated with antibody and T-cell responses. Science Immunology.

[27] Janice Oh HL, Ken-En Gan S, Bertoletti A, Tan YJ (2012). Understanding the T cell immune response in SARS coronavirus infection. Emerg. Microbes Infect..

[28] Tseng CT (2012). Immunization with SARS coronavirus vaccines leads to pulmonary immunopathology on challenge with the SARS virus. PLoS ONE.

[29] Iwata-Yoshikawa N (2014). Effects of Toll-like receptor stimulation on eosinophilic infiltration in lungs of BALB/c mice immunized with UV-inactivated severe acute respiratory syndrome-related coronavirus vaccine. J. Virol..

[30] Bolles M (2011). A double-inactivated severe acute respiratory syndrome coronavirus vaccine provides incomplete protection in mice and induces increased eosinophilic proinflammatory pulmonary response upon challenge. J. Virol..

[31] Yasui F (2008). Prior immunization with severe acute respiratory syndrome (SARS)-associated coronavirus (SARS-CoV) nucleocapsid protein causes severe pneumonia in mice infected with SARS-CoV. J. Immunol..

[32] Wang Q (2016). Immunodominant SARS coronavirus epitopes in humans elicited both enhancing and neutralizing effects on infection in non-human primates. ACS Infect. Dis..

[33] Agrawal AS (2016). Immunization with inactivated Middle East Respiratory Syndrome coronavirus vaccine leads to lung immunopathology on challenge with live virus. Hum. Vaccin Immunother..

[34] Luo F (2018). Evaluation of antibody-dependent enhancement of SARS-CoV infection in Rhesus Macaques immunized with an inactivated SARS-CoV vaccine. Virol. Sin..

[35] Qin E (2006). Immunogenicity and protective efficacy in monkeys of purified inactivated Vero-cell SARS vaccine. Vaccine.

[36] Roberts A (2010). Immunogenicity and protective efficacy in mice and hamsters of a beta-propiolactone inactivated whole virus SARS-CoV vaccine. Viral Immunol..

[37] Deng Y (2018). Enhanced protection in mice induced by immunization with inactivated whole viruses compare to spike protein of middle east respiratory syndrome coronavirus. Emerg. Microbes Infect..

[38] Zhang N (2016). Identification of an ideal adjuvant for receptor-binding domain-based subunit vaccines against Middle East respiratory syndrome coronavirus. Cell Mol. Immunol..

[39] Luke T (2016). Human polyclonal immunoglobulin G from transchromosomic bovines inhibits MERS-CoV in vivo. Sci. Transl. Med..

[40] Darnell ME (2007). Severe acute respiratory syndrome coronavirus infection in vaccinated ferrets. J. Infect. Dis..

[41] Smith TRF (2017). Development of an intradermal DNA vaccine delivery strategy to achieve single-dose immunity against respiratory syncytial virus. Vaccine.

[42] Yan J (2007). Enhanced cellular immune responses elicited by an engineered HIV-1 subtype B consensus-based envelope DNA vaccine. Mol. Ther..

[43] Bagarazzi ML (2012). Immunotherapy against HPV16/18 generates potent TH1 and cytotoxic cellular immune responses. Sci. Transl. Med..

[44] Caly, L. et al. Isolation and rapid sharing of the 2019 novel coronavirus (SARS-CoV-2) from the first patient diagnosed with COVID-19 in Australia. *Med. J. Aust.* (2020). 10.5694/mja2.50569 PMC7228321 32237278

[45] Huang PS (2011). RosettaRemodel: a generalized framework for flexible backbone protein design. PLoS ONE.

