# Supplementary material for: Immunogenicity of a DNA vaccine candidate for COVID-19
Source: Nat Commun. 2020 May 20;11:2601. doi: 10.1038/s41467-020-16505-0 (PMC7239918; doi:10.1038/s41467-020-16505-0)
Supplement: Supplementary file 1 — Supplementary Information [file 41467_2020_16505_MOESM1_ESM.pdf]

Supplementary Figures.

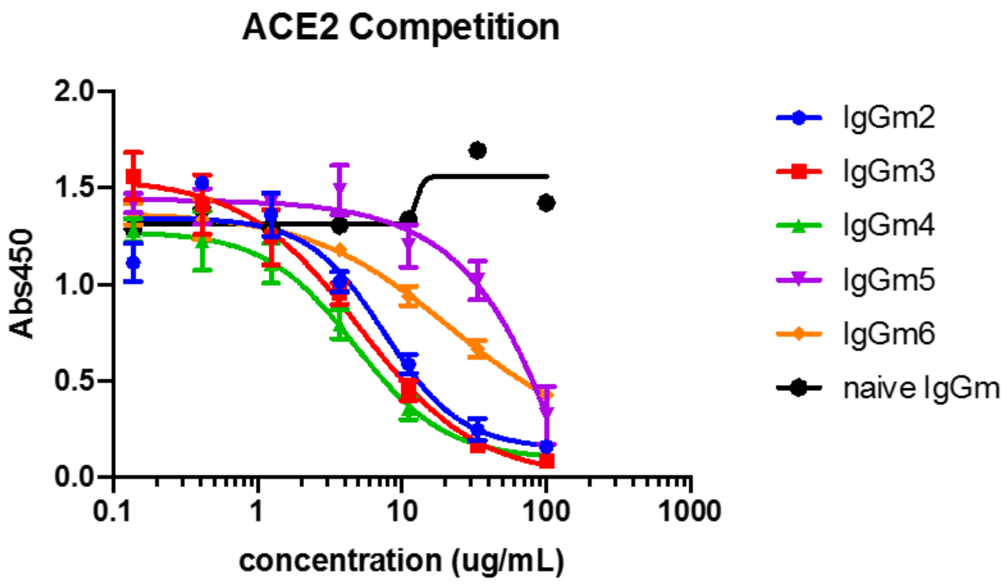

Supplementary Figure 1: IgGs purified from n=5 mice day 14 post second immunization with INO-4800 show competition against ACE2 receptor binding to SARS-CoV-2 Spike protein compared to pooled naïve mice IgGs (black). Naïve mice were run in a single column. Vaccinated mice were run in duplicate. If error bars are not visible, error is smaller than the data point.

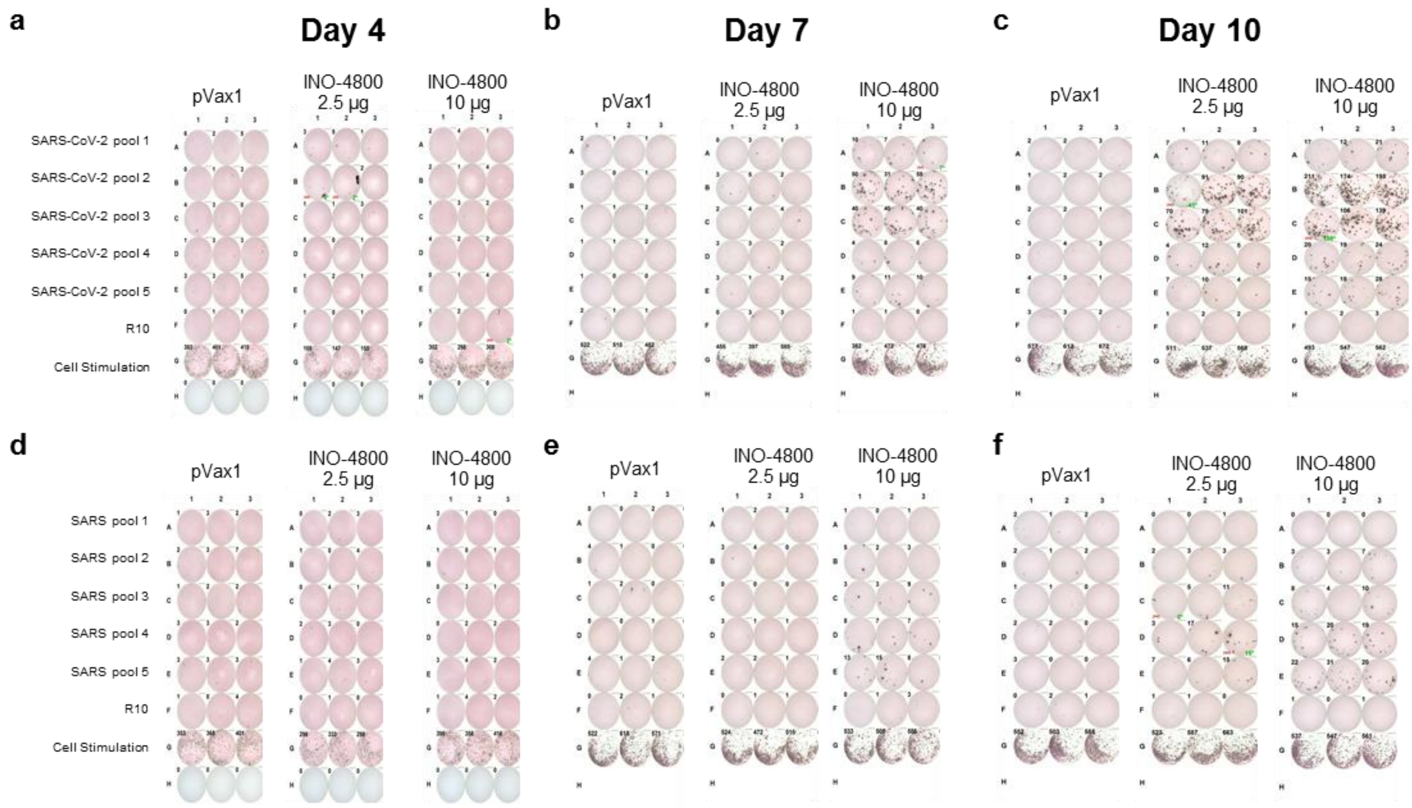

Supplementary Figure 2. ELISpot images of IFN-γ+ mouse splenocytes after stimulation with SARS-CoV-2 and SARS antigens. Mice were immunized on day 0 and splenocytes harvested at the indicated time points. IFNγ-secreting cells in the spleens of immunized animals were enumerated via ELISpot assay. Representative images show SARS-CoV-2 specific (a,b,c) and SARS-CoV-specific (d,e,f) IFNγ spot forming units in the splenocyte population at days 4, 7, and 10 post-immunization. Images were captured by ImmunoSpot CTL reader.

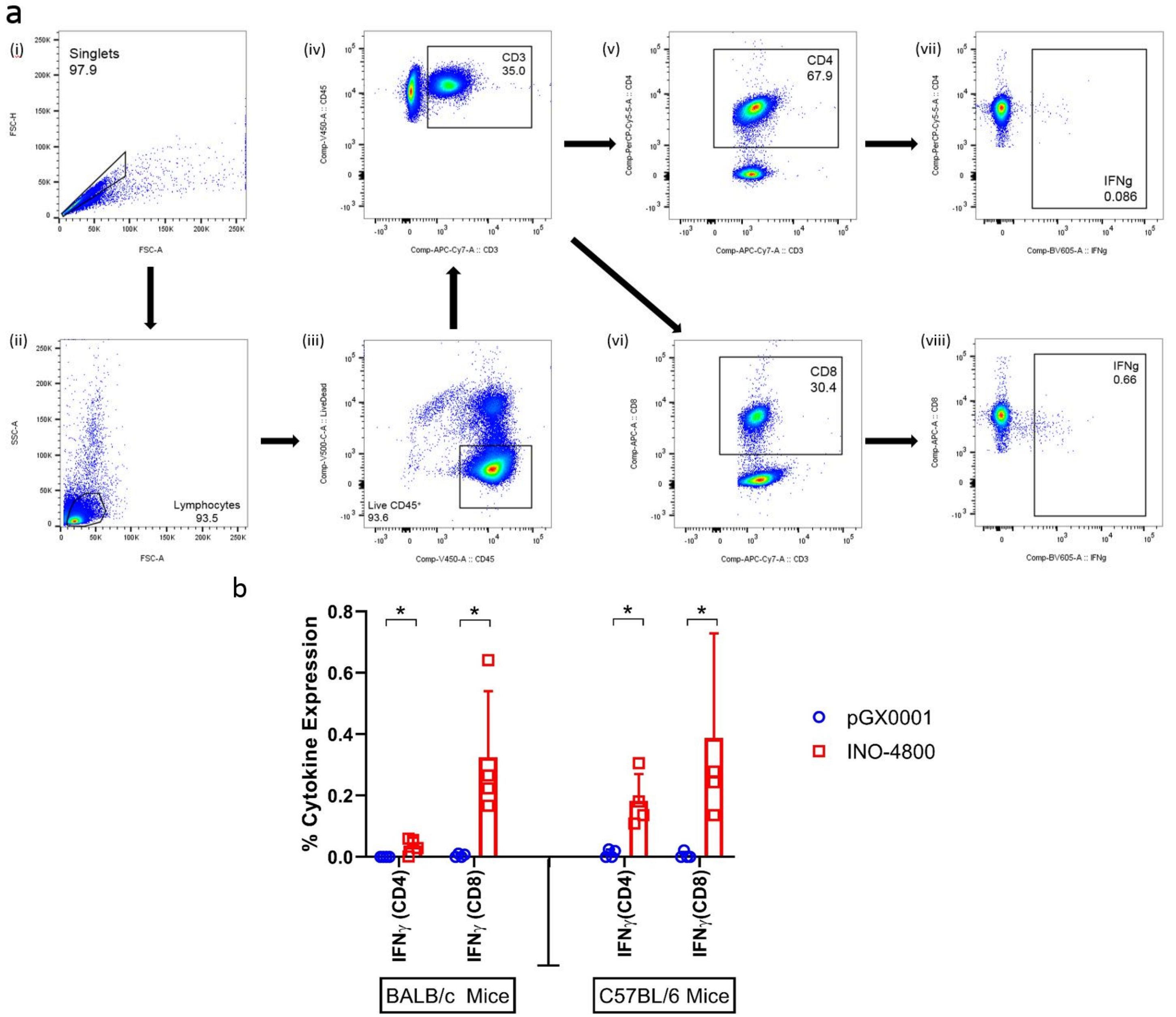

Supplementary Figure 3. Flow cytometric analysis of T cell populations producing IFN-γ upon SARS-CoV-2 S protein stimulation. Splenocytes harvested from BALB/c and C57BL/6 mice 14 days after pVAX or INO-4800 treatment were made into single cell suspensions. The cells were stimulated for 6 hours with SARS-CoV-2 overlapping peptide pools. (**a**) CD4+ and CD8+ T cell gating strategy; singlets were gated on (i), then lymphocytes (ii) followed by live CD45+ cells (iii). Next CD3+ cells were gated (iv) and from that population CD4+ (v) and CD8+ (vi) T-cells were gated. IFNγ+ cells were gated from each of the CD4+ (vii) and CD8+ (viii) T-cell populations. (**b**) The percentage of CD4+ and CD8+ T cells producing IFNγ is depicted. Bars represent mean +SD. 4 BALB/c and 4 C57BL/6 mice were used in this study. * $p < 0.05$, Mann Whitney test.
