# Supplementary material for: Immunogenicity of a DNA vaccine candidate for COVID-19
Source: Nat Commun. 2020 May 20;11:2601. doi: 10.1038/s41467-020-16505-0 (PMC7239918; doi:10.1038/s41467-020-16505-0)
Supplement: Supplementary file 3 — Description of Additional Supplementary Files [file 41467_2020_16505_MOESM3_ESM.pdf]

Description of Additional Supplementary Files

File Name: Supplementary Data 1
Description: Percent Identity Distance Heatmap between Two SARS-Cov2 Spike Protein Amino Acid Sequences from GISAID.

Whole genome sequences of SARS-Cov2 available on GISAID up to date March 30th were downloaded. Spike protein genome sequences of each reported isolate was extracted via a pearl script with primer sequences “ATGTTTGTTTTTCTTGTTT” (Forward) and “GGAGTCAAATTACATTACACATAA” (Backward). All spike protein sequences were then translated and aligned to Wuhan-Hu-1 using the software Geneious Prime (Clustal Omega 1.2.3 alignment algorithm). Percent Identity Distance between each two isolate sequences was calculated and presented as a cell value in the table. Wuhan-Hu-1’s Percent Identity in the population was calculated by taking the average of its Percent Identity Distance to every other isolate.

File Name: Supplementary Data 2
Description: Source data for BALB/c T cell responses shown in Figure 9 a-c. Spot forming cell (SFC) values for individual mice in response to SARS-CoV-2, SARS, and MERS peptide pools are shown for the indicated collection time points. T cell responses were measured by IFN-γ ELISpot in splenocytes stimulated for 20 hours with overlapping peptide pools spanning the SARS-CoV-2, SARS-CoV, or MERS-CoV Spike proteins.
